# The application of bacteriophage to veterinary and One-Health medicine—a road map

**DOI:** 10.3389/fmicb.2025.1725071

**Published:** 2026-01-29

**Authors:** Robert J. Atterbury, Adriano M. Gigante, Matti Jalasvuori, Robert Lavigne, Catherine Schouler, Valeria Mariano, Paul Barrow

**Affiliations:** 1 School of Veterinary Medicine and Science, University of Nottingham, Loughborough, United Kingdom; 2 Department of Biological and Environmental Sciences, University of Jyväskylä, Jyväskylän, Finland; 3 Laboratory of Gene Technology, KU-Leuven, Leuven, Belgium; 4 French National Institute for Agriculture, Food, and Environment (INRAE) Val de Loire UMR ISP, Nouzilly, France; 5 STAR IDAZ Secretariat, Science Department, World Organisation for Animal Health, Paris, France; 6 School of Veterinary Medicine, University of Surrey, Surrey, United Kingdom

**Keywords:** AMR, antimicrobial-resistant, bacteriophages, one-heath medicine, phage

## Abstract

The STAR-IDAZ international research consortium established a working group on Alternatives to Antimicrobials to explore various approaches for reducing our reliance on antimicrobials. These included bacteriophages, activating the immune system and manipulating the microbiome. The sub-group investigating bacteriophages have developed a road map for the application of phages in a One Health context. We present this roadmap here, in review format, along with a discussion of how phages may be combined with other therapies.

## Bacteriophages used for infection control—a brief history

The use of bacteriophages (phages) for treating bacterial infections has now been considered for more than a century—longer than the use of traditional antibiotics. Both have had a mixed history; bacteriophages because of issues related to the practicality of their use and intellectual property, and antibiotics because of problems arising from the development of resistance. Phages are now considered as having real potential for dealing with antimicrobial-resistant (AMR) bacterial pathogens. Antimicrobials are becoming less effective, to the extent that a small, but increasing, number of bacterial infections are now completely refractory to antimicrobial treatment. Phages as bactericides are now being considered for application in several fields besides infection control, including use in agriculture and microbiome modulation (García-Cruz et al., 2023).

It is somewhat ironic that of the two independent discoverers of bacteriophage activity, Twort and d’Herelle, the scientist who had the imagination to explore their use for controlling infections was not a professional microbiologist. It was d’Herelle who explored the use of phages against several infections *in vivo*, including *Salmonella* Pullorum in chickens (d’Herelle, 1921) and human dysentery (d’Herelle, 1926). d’Herelle’s close association with George Eliava in Georgia led to Stalin’s investment in this technology with an institute (George Eliava Institute)^1^ dedicated to phage use with promulgation of the idea of phage therapy across the Soviet bloc. Routine medical applications there continued even after 1989, and right up to the present day,^2^ currently supported by the FAO and EU for its work on veterinary pathogens.^3^

Early work was also carried out in the West with retrospective concerns over the quality of the work itself. A trial in the 1930s assessing efficacy against cholera involved simply pouring a phage preparation into a drinking water well and counting the number of cases of cholera in the immediate vicinity. It must be remembered that at that time nothing was known about the real nature of bacteriophage, other than that it was a replicating lytic principle which passed a bacteriological filter. Because of this, there was limited understanding of the criteria required for selecting the most effective phage for application. The basis of bacterial pathogenesis, the nature of the interaction between susceptible bacteria and phage and therefore the scope for phage application was too poorly understood on which to build a new therapy. Having said that, d’Herelle was nominated for a Nobel prize several times, yet it was never conferred.

The introduction of antimicrobials from the 1940s led to further work on phage being largely discontinued in western countries. By contrast, phage therapy in Eastern European countries and elsewhere continued, although it became apparent later that few trials contained experimental controls, and efficacy was often difficult to assess with frequent use of combinations of phage with other treatments, including antimicrobials.

The situation in the West changed in the 1980s when Smith and his colleagues in the UK demonstrated, with some well controlled experimental work, that lytic phages could be highly effective in preventing and treating *Escherichia coli* septicaemia in mice, poultry and calves (Smith and Huggins, 1982; Barrow et al., 1998) and diarrhoea in calves, pigs and lambs induced by enterotoxigenic strains of *E. coli* (ETEC, Smith and Huggins, 1983; Smith et al., 1987). Phages used against septicaemia were generally more effective than antimicrobials (Smith and Huggins, 1982) and could be applied even after clinical signs began to appear (Smith and Huggins, 1982; Barrow et al., 1998). It was apparent from these studies that phage did not eliminate the bacteria completely from the host but reduced them to a level not associated with clinical disease, relying on innate and acquired immunity to remove the remaining bacteria.

The demonstration of the high potential efficacy of lytic phages led to an explosion of further work as well as proposed frameworks for the use of phage therapy (Kutter and Sulakvelidze, 2005; Merabishvili et al., 2009; Pirnay et al., 2018; Gigante and Atterbury, 2019; Hatfull et al., 2022; Strathdee et al., 2023; Pirnay et al., 2024). Some of the work was effective, some not and some with phage used inappropriately. Application is most effective under conditions comparable to those that promote optimal phage growth *in vitro*. These include the surface of the small intestinal and other mucosa and skin (similar to a bacterial lawn) and blood, the small intestine and cerebro-spinal fluid (analogous to liquid culture with a similar degree of mixing). They should thus be effective in treating septicaemias and some enteric infections, reducing infection of burns and reduce skin colonisation by bacteria including those that are AMR. Phages can also be applied under conditions which are not reliant on phage replication for a therapeutic effect. When applied at a very high multiplicity of infection (MOI), phage may disrupt the bacterial membrane potential, rapidly resulting in cell death. This approach has been used in carcass and surface decontamination and food treatment.

**Figure 1:**
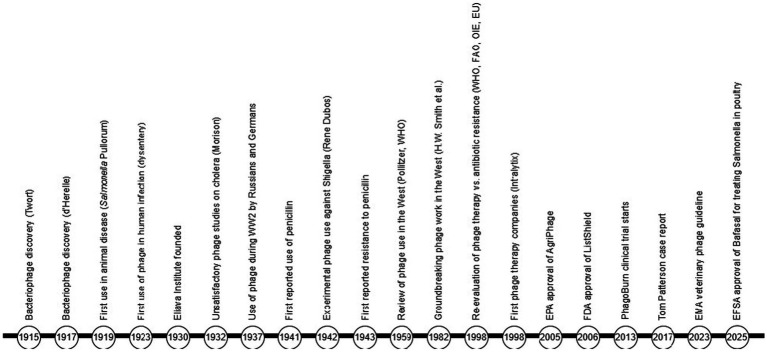
A timeline of bacteriophage use and application in clinical and veterinary medicine (European Commission, 2025; European Medicines Agency, 2023; Environmental Protection Agency, 2005; Food and Drug Administration, 2006; Patterson, 2017; Rose et al., 2014).

Despite extensive research (>13,979 entries on PubMed just for “phage and therapy” as the search term in December 2025); the number of successful applications is relatively small (867 entries if “success” is added to the search) and the number involving food treatment is just 2,428. Some have been unexpectedly successful (Dedrick et al., 2019) while others less so, even if success was expected (Jault et al., 2019). Notwithstanding the mixed quality of some published reports in this field (Uyttebroek et al., 2022), there are sufficient high-quality reports of successful phage therapy to warrant its serious consideration as an alternative treatment, particularly in the context of the growing AMR threat. The long timeline from the discovery of bacteriophages through to the establishment of the first western company, specifically aimed at developing phage for clinical use, is shown in Figure 1.

## Antimicrobial resistance, an existential one health problem and attempts to ameliorate AMR

AMR is now reaching the stage where it is regarded as a major global public health threat. Fleming recognised that resistance would emerge soon after introduction of an antibiotic and this has always been the case. Strains of *S. aureus* became resistant to penicillin rapidly and have continued to increase the range of antibiotics to which they are resistant, mainly mediated by transduction (Lindsay, 2014). In animals the use of antibiotics for treatment and prophylaxis against enteritis in intensively reared calves and for growth stimulation of livestock in Europe from the 1950s to the 1970s contributed to multi-resistance in *E. coli*, *Salmonella* and related enterobacteria (Anderson, 1968). This situation continues to deteriorate because of regulated and unregulated treatment, prophylaxis and meta-phylaxis in livestock today and continued smaller scale use in plant rearing (Velazquez-Meza et al., 2022). Growth stimulation using antibiotics is still practised in various parts of the world. AMR bacterial strains are isolated increasingly from wild-life and the environment and their impact on One-Health with the inter-relationships between the health of man, animals and the environment (Larsson et al., 2023) has been emphasised by the WHO, FAO, WOAH (founded as OIE) and UNEP.^4^ While the environment contributes antibiotic resistance genes, the primary concern should be addressing the main source of multi-resistance: antimicrobial-resistant commensal and pathogenic bacteria in humans and livestock, which result from the use, overuse, and misuse of antimicrobials.

Plasmid-mediated resistance involving transposons and integrons enables bacterial strains to transfer their resistance to related bacteria and accumulate AMR genes, becoming highly multi-resistant (Harris et al., 2023). Plasmid-mediated self-transmissibility is complicated by the incorporation of virulence genes and multiple replicons in individual plasmids. Considerable research efforts, over many years, have explored pharmaceutically-induced instability of AMR-encoding plasmids (Williams and Hergenrother, 2008; Baquero et al., 2011; Buckner et al., 2018; Getino and de la Cruz, 2018), without demonstrable success *in vivo*. The success of AMR plasmids is indicated by the global spread of individual AMR *E. coli* clones including ST 131 and its derivatives, harbouring F plasmids that have shaped their evolution (Pitout and DeVinney, 2017). Increasingly, clinically important pathogens, including the so-called ESKAPE pathogens are becoming resistant to the CIAs (Critically Important Antibiotics) normally restricted to the treatment of serious human diseases (Miller and Arias, 2024).

The plasmids on which antibiotic resistance genes are carried are classified into Incompatibility (Inc) groups, a small number of which dominate human and veterinary medicine (Carattoli, 2009; Rozwandowicz, et al., 2018). Classification is based on the origin of replication, with some plasmids having more than one and thus belonging to more than one Inc. group. Most predominant AMR plasmids are self-transmissible, with plasmid transfer encoded by large transfer gene clusters and contact with potential recipient bacteria mediated via proteinaceous filamentous sex pili. However, the production of sex pili at the bacterial surface renders them susceptible to attack by classes of phages which attach specifically to these organelles (*vide infra*). Because of this, transmissibility in most plasmids is generally repressed by varying orders of magnitude with sometimes very few cells being able to transfer the plasmid in any one culture at any time. This balances self-transmissibility with resistance to environmental phages.

Society has reached a critical juncture (Figure 1), recognised for many years by international institutions including the WOAH^5^ and WHO^6^ (Report, 2015; Report, 2018; Willemsen et al., 2022). They recognise the importance of improved and rapid diagnosis, improved vaccination and reduction in use of antibiotics including the complete withdrawal of their use for growth stimulation. However, these measures will not lead to a rapid reduction in AMR in bacterial strains which are already resistant, and international institutions have called for research into novel approaches to controlling AMR infections, including the use of bacteriophage (Report, 2014; Pelfrene et al., 2016; OECD, 2022).

## A road map for future exploration and implementation

It remains the case that for more extensive application of phage for control of AMR bacteria, several imponderables need to be taken into consideration and, ideally, resolved. The STAR-IDAZ International Research Consortium^7^ is a global initiative to address the coordination of research programmes at the international level in the area of animal health and in particular infectious animal diseases including zoonoses. It has explored key strategies to reduce AMR by implementing research on Alternatives to Antimicrobials (ATA). To enhance international collaboration on focused priorities, working groups of international experts were convened to develop research roadmaps on ATA with the aim of identifying the critical knowledge gaps to be addressed to deliver a range of possible ATA (e.g., vaccines, immunomodulators, PRR agonists, chemicals, phytochemicals, microbiota, and phage technologies), targeting the pathogen or the host or both. Bacteriophage applications against AMR were analysed in terms of opportunities and challenges and identifying key targets for further research which are necessary for more widespread use in human and animal medicine. This review is the result of that exploration.

**Figure 2:**
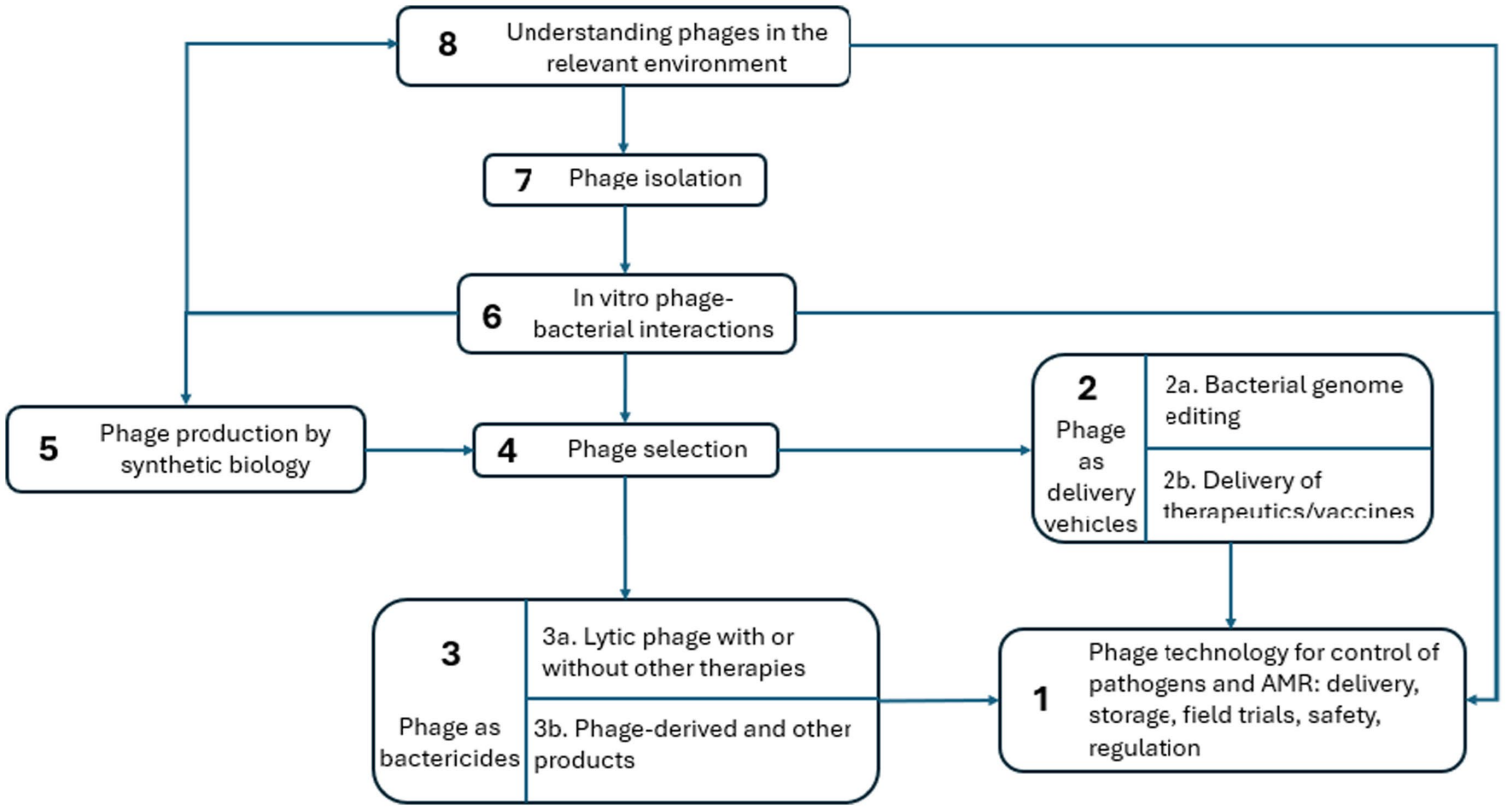
STAR-IDAZ roadmap showing the phage therapy development nodes which are referenced in the following summary.

Given the existential nature of the AMR threat, and the extent to which international institutions favour the further exploration of bacteriophage and its implementation, several issues remain which must be taken into account before phages are ready to be used. These include improved understanding of phage selection, phage-host interactions, applications in different diseases/species, One Health impacts and regulation. Additional challenges remain concerning genetically engineered phage, and how best to manage phage in combination with other therapeutic approaches. The STAR-IDAZ group has incorporated these points into a roadmap (Figure 2).

## Key sections of the roadmap

The phage technology roadmap shows (Node 1) the intended outcome: phage technology for pathogen control, ideally presented in the form of a target product profile. The roadmap describes the research steps needed to achieve this goal. Each node or ‘Lead’ includes five areas that highlight the knowns and unknowns for the issue in question: (i) research question; (ii) challenge; (iii) solution routes; (iv) dependencies; and (v) state of the art. Each Lead has an overarching question and/or statement of intent with supporting information that is designed to focus research efforts (Entrican et al., 2021). The various nodes are mutually interactive since data collected from *in vitro* studies can lead to a better understanding of the interactions with the target pathogen *in vivo* and to the conditions required for delivery. The summary of the roadmap is presented in Table 1.

**Table 1:** Summary of roadmap: questions, challenges and solutions.

Node number and title	Questions to be answered	Challenges	Solutions
8. Understanding phages in the relevant environment	Can we define the conditions under which phage interact with target bacteria *in vivo*?	Identifying the exact conditions of interest and practical significance which will inform the delivery of a final product in Node 1.	Use or develop realistic *in vivo* models. Use knowledge gained from Node 6.
7. Phage isolation	Can we maximise isolation of phage relevant for clinical use from a variety of environmental sources? Can we prepare pure high titre phage preparations?	Crowding out of potentially useful and new phages by larger numbers of other phages. Need to develop isolation techniques for new environmental phages.	Enrichment with single or mixtures of potential host bacterial strains. Development of wide variety of culture conditions for environmental phages. Review all available methods for high titre phage production.
6. *In vitro* biology of phage-bacterial interactions	Can we define precisely and quantitatively the dynamics of phages selected for potential use from Node 7, to mimic the conditions in Node 8 and inform on final use in Node 1?	Enabling the definition of conditions for the study of *in vitro* dynamics for a potentially large number of phages under a range of conditions. Studying large numbers of phages under a variety of conditions.	Use of low- and high-throughput systems.
5. Phage production by synthetic biology	Can we synthesise phage *de novo* to eliminate dangerous genes and modulate host range.	Can we reproducibly synthesise a consistent genome sequence from RNA phages.	Biosynthesis followed by reverse transcriptase and sequencing.
4. Phage selection	Using the refined criteria for selection from Nodes 5, 6 and 8 can we select the potentially most effective phages.	Apart from burst size/latent period, identifying criteria which will lead to selecting phages of maximum effectiveness under the projected *in vivo* conditions. Identifying dangerous genes	Development and use of suitable *in vitro* and *in vivo* models to evaluate candidate phages. Genome sequencing to identify dangerous genes for removal in Node 2a.
3.	Phage and their products as bactericides		
3a. Lytic phage with or without other therapies	Can we identify the conditions under which phages can be effective by their lytic activity alone or in the presence of other agents such as antibiotics and disinfectants.	Standardisation of *in vitro* protocols and *in vivo* models for quantitative evaluation.	Further evaluation of combined therapy with phages, especially with antibiotics and disinfectants.
3b. Phage-derived and other products	Can we identify the conditions under which phage-derived and similar products can be effective by their lytic activity.	With the exception of endolysins, there is insufficient information and research on the use of phage products in appropriate *in vivo* models and *in vitro*.	Further *in vitro* evaluation for tailocins and related products and further *in vivo* work, clinical and with animal models, on the use of endolysins.
2.	Phage as delivery vehicles		
2a. Bacterial genome editing	Can we exploit molecular biological methods to expand phage host-range, delete harmful phage sequences and introduce sequences harmful to the target bacterium.	Selection of appropriate *in vivo* models where modified phages and attenuated bacterial strains can be assessed for changes of phenotype.	Experimental work using *in vitro* and *in vivo* models to assess practicality and safety of gene-edited phages.
2b. Delivery of therapeutics/vaccines	Can we use phages to deliver pharmaceutically active molecules and introduce into phage genomes nucleic acid-based products to modulate phage surface epitopes to immunize the host or be used for diagnostic purposes.	Appropriate immunological assays *in vitro* and *in vivo* are already available. Appropriate *in vivo* models for immunity and pharmacodynamics studies.	Experimental work using *in vitro* and *in vivo* models to assess practicality and safety of immunomodulated phages and the *in vivo* effect of pharmaceutically active molecules.
1. Phage technology for control of pathogens and AMR: delivery, storage, field trials, safety, regulation	Can we develop a clinical package (i) optimising storage and delivery, (ii) introducing standards for animal (field) trials, and (iii) develop a system for fast-track regulatory approval for phages, phage banks and magistral phage.	Insufficient work on storage, field trial standardisation and overcoming the hurdles associated with regulation and intellectual property.	Further work on (i) storage, encapsulation and liposome delivery, (ii) establishment of criteria for animal trials assessment, and (iii) liaison with international bodies and governments to ensure that regulation does not hinder further development.

The interactive phage research roadmap, briefly described below, is available for detailed consultation on the STAR-IDAZ website.^8^

### Node 8—Understanding phages in the relevant environment

The starting point for the roadmap involves defining the target bacteria based on the specific disease/infection/pathogenesis scenario together with an understanding, as profound as possible, of the colonisation/pathogenesis conditions in the eukaryotic host. The research question and challenge here is to understand wild phages and their behaviour in relevant environments, including animal reservoirs. This is fundamental to identifying those phages with appropriate ecological fitness and applicability in real-world conditions where interactions with different environmental factors is the norm.

An understanding of the *in vivo* interaction will arise out of knowledge of the specific *in vivo* conditions under which the phage interacts with the target pathogen supported by the information arising from the more controlled *in vitro* interactions elucidated in Node 6. The combination of the information from both these nodes will then feed into Node 1 which will define the conditions under which the phage will be delivered for the purposes of controlling the target pathogen identified in Node 8.

The conditions under which the phage and host were originally isolated, may differ from those required for phage therapy since they may not be applied in the host in a similar internal environment. For example, intestinal or skin phages isolated from sewage might be applied to treat septicaemia. A future opportunity may arise from the burgeoning interest in the wide variety of unusual phages found in sea water and other environments which seem to differ hugely from those that are normally associated with human and animal pathogens. Little is known about them and it remains to be seen whether their genomes and behavioral patterns have anything to contribute to their use against particular pathogens and to AMR.

Acquisition of this information may be dependent on the availability/development of relevant *in vivo* infection models. Apposite infection models involving phage therapy have been in use for a number of years in domestic and laboratory animals for bacterial gastroenteritis, caused by *E. coli* pathotypes (Smith and Huggins, 1983), *Salmonella* (Atterbury et al., 2007) and *Campylobacter* (Loc Carrillo et al., 2005). Bacterial septicaemia may also be induced easily in mice, poultry and calves (Smith and Huggins, 1982; Barrow et al., 1998). These are also highly relevant to complex urinary tract infections (Morgan et al., 2025) which may involve progress to pyelonephritis and sepsis. Lytic phages have already demonstrated efficacy under these *in vivo* conditions. Models of respiratory, skin and other infections have been established for several bacterial pathogens some of which may be extrapolated to the human condition (Malik et al., 2017). All these ecological niches have direct relevance to bacterial targets which are AMR.

### Node 7—Phage isolation

Once the target pathogen, serotype and strain has been defined, suitable phages must be sought. Unmodified, wild phages can usually be isolated from environments in which the host bacteria are routinely present. Depending on the target bacterium, these would typically include clinical samples (intestinal samples, skin, mucus, sputum, respiratory tract etc.) from infected patients or animals. In many cases and not surprisingly, phages associated with a variety of human or animal bacteria can all be isolated from raw sewage, farm waste, market drains etc. The diversity in sample sources increases the likelihood of finding phages effective against a wide range of AMR bacteria. Future opportunities may also exist from sourcing phages from environments not directly associated with the clinical pathogens, such as soil, sea water etc.

Isolation of wild bacteriophages has been routine for several decades. Environmental samples may be used directly without an enrichment step if phages are present in sufficient numbers and the bacterial host species divides rapidly, otherwise, samples may require centrifugation and filtration (Van Twest and Kropinski, 2009) followed by a broth enrichment stage. Although only one bacterial strain is generally used, to isolate phages, particularly RNA phages, with broader host range, this enrichment step might involve mixing with cultures of several strains. Moreover, as phages targeting bacterial virulence factors or sex pili as receptors can benefit phage therapy, the bacterial host may be grown in specific media facilitating their expression. The phage-host culture will require further centrifugation and filtration. Phage detection/isolation in/from the filtrate is usually performed using plaque assays (Nair et al., 2021; Daubie et al., 2022). Individual clear plaques are then serially purified and amplified on the target bacterial host.

To increase the number of phage strains available for use and to establish phage biobanks (*vide infra*), citizen science projects have been involved. For example, in the UK, over 1,000 phages against the WHO priority pathogens were isolated from water- and waste-water samples in the citizen phage library project (Fletcher et al., 2024). The phages isolated were tested against various AMR bacterial strains to determine their spectrum of activity, a vital step to select phages with broad-spectrum activity.

Use in therapy is dependent on bacterial cell debris and LPS being removed completely from phage suspensions. Protocols based on cross-flow ultracentrifugation and LPS-affinity or ion-exchange chromatography have been described (Adriaenssens et al., 2012; Luong et al., 2020). Further production and purification of high-titre phage suspensions will require sucrose density gradient ultracentrifugation as CsCl is toxic to cells (Kosznik-Kwasnicka et al., 2023). Other strategies rely on affinity purification approaches, tailored towards individual phages.

### Node 6—*in vitro* biology of phage-bacterial interactions

Successful development of phage technology cannot progress without sound knowledge of basic phage biology, including the phage life cycle, dynamics and host receptor binding in simple and also complex microbial communities for phages.

The life cycle of some phages has been well understood for several decades, both at the biological and molecular level, to obtain a more profound understanding of bacterial and phage genetics. Much remains to be done to fully understand existing and new phages which show potential for medical/veterinary application. We also have virtually no understanding of the dynamics of the huge number of phages which are increasingly isolated from the environment and which might be used clinically. Phages isolated from the marine environment might, for example, be used against *Vibrio* species such as *V. cholerae*, *V. vulnificus* etc.

Approaches can be adopted to target bacterial pathogens with phages for which surface virulence determinants are the phage receptors. For this reason, the identity and nature of phage receptors should be determined.

Phages are considered potentially effective when they interact with the bacterial host under conditions which reflect the optimal conditions for multiplication *in vitro*, including attachment to the host bacterial cell. Most *in vivo* studies, both experimental and therapeutic/prophylactic, have involved enteritis, septicaemias, and infections of the skin, urinary tract and cerebrospinal fluid which favour contact between bacterial host and phage. Establishment of *in vitro* models for some of these *in vivo* environments would benefit phage selection (Node 4) and the development of delivery protocols (Node 1). There is a suggestion that phage may be less effective against intracellular pathogens although this has also not been modelled *in vitro*.

Although lysis from without using high Multiplicity of Infection (MOI) has been demonstrated to be effective in reducing carcass or surface bacterial contamination, the optimal conditions for phage attachment are still poorly understood.

### Node 5—phage production by synthetic biology

Questions arise about the variable genetic content of some phages. Although unwanted genes and genomic components can be removed from phage genomes this can be arduous. An alternative is phage production through synthetic biology, which can provide scalable, reproducible, and precisely engineered phages, combining synthesizing entire phage genomes in combination with *in vitro* assembly. This is likely to be most economical and practical for small RNA viruses with rescue in the appropriate bacterial host. There is already considerable experience with the synthesis of polio virus using reverse transcriptase (Cello et al., 2002), the small and larger DNA phages (Shin et al., 2012) and the 11.1 kb Vesicular Stomatitis Virus (VSV) (Moles et al., 2024). This approach has been used for functional and clinical studies (Chari and Church, 2017; Huang et al., 2019) and improving viral vectors (Nie et al., 2024; LeNouën et al., 2017) and viral vaccines (Thi Nhu Thao et al., 2020).

Sequence modulation may also be introduced in due course using AI to explore expansion of host range. The combination of such approaches with emerging insights into phage biology will enable more engineering freedom, through a careful design-build-test-learn SynBio strategy tailored to phage genomes.

The population structure of phage genomes is also relevant. For some studies RNA phages may be preferred for specific therapies and the consensus genome sequence will be used for any genome synthesis studies but it is well understood that considerable variation in sequence between individual phage particles and phenotype will occur both *in vitro* and *in vivo*.

One advantage that synthesis of bacteriophages has over eukaryotic viruses is fewer ethical issues (Lipsitch and Galvani, 2014).

### Node 4—Phage selection

Developing methods for selection of suitable, stable and robust phages from natural or engineered libraries, ensuring specificity and effectiveness against target pathogens is considered in Node 4. Apart from the generic criteria of burst size and latent period mentioned above, measured crudely as rate of lysis (Smith, 1982), specific criteria may be selected depending on what is known about related better-understood phages and also on what is required of the phage in relation to the bacterial host and the infection it produces. However, the *in vivo* environment, involving factors such as inactivating serum proteins or the presence of complex microflora, may affect phage activity and has not been studied in any depth. The choice of many criteria by which large phage collections may be screened is dependent on having the knowledge of the nature of the interaction between bacterium and phage (see Nodes 6 and 8). The full information of the phage biology is most completely represented by its genome and this information may be used to identify genes or elements which are desirable or undesirable for practical use. This may be further augmented by RNA-seq analysis of gene expression, which provides information about phage replication and response of the bacterial host. The latter may be particularly important in the case of engineered phage, where unexpected gene expression profiles may reduce efficacy of therapy or even damage animal health. It may also reveal interactions between therapeutic phage and endogenous prophage (Finstrlová et al., 2022). To this can be added information on effectiveness including interactions with the host immune system which may have a key role to play in therapeutic efficacy, and of which relatively little is known (Krut et al., 2018). High throughput systems may be used for *in vitro* screening, measuring changes in culture optical density using equipment such as the Biocore (BioDesign). Taken together, these datasets can very quickly become vast and unwieldy but may be amenable to machine learning and AI which can rapidly identify patterns in the data which may be useful in optimising phage for clinical applications (Keith et al., 2024).

The efficiency of phage infection must be measured on a range of different bacterial strains, likely to be involved in an infection, to quantify the phage’s effectiveness. Some staphylococcal phages already appear to have broad host range (Feng et al., 2021; Göller et al., 2021). The assessment helps in selecting the most potently lytic phages for therapeutic use. To evaluate the potential for bacteria to develop resistance to the phage, serial passage experiments should be conducted.

It is assumed that (i) successful phages will be highly active *in vitro* and, by extension, *in vivo* and also that (ii) application will be optimal in clinical conditions where the opportunity for contact between phage and bacterial host is also optimal (Barrow and Soothill, 1997). However, whether this is exclusively the case for all potentially useful phage remains to be seen.

During the selection process there is an absolute necessity for the removal from the phage genome of any potentially harmful genes which may be harmful in themselves or may regulate the expression of bacterial genes, particularly those associated with virulence. Phages, whether naturally lytic or temperate, have the capacity to acquire and transfer bacterial genes which may have potentially harmful consequences. Phage genes associated with chromosomal integration must be deleted by genome manipulation or by *de novo* phage genome synthesis.

### Node 3—Phage and their products as bactericides

Lytic phages have been used alone but also in combination with antibiotics or, for topical use, disinfectants. Phage components are also being explored for their bactericidal properties.

#### Node 3a—lytic phage with or without other therapies

Lytic phages may be used directly against target bacteria, exploiting their ability to induce host cell lysis. With members of the *Enterobactericeae*, specificity is linked to the surface receptors which in many cases are lipopolysaccharide or capsular material. This can limit the extent to which phage can be applied generically against different serovars of the same bacterial species. This is not necessarily the case for other pathogens which may be less genomically diverse, including *Staphylococcus aureus* (Feng et al., 2021; Göller et al., 2021) where phage have been isolated with a broader host range increasing their potential applicability.

Lytic phage may also be used to kill bacteria by non-specific lysis/lysis from without by applying them at a very high MOI. This has been used experimentally for skin decontamination (Goode et al., 2003; Atterbury et al., 2003; Hooton et al., 2011) and to reduce bacterial contamination of food surfaces including cheeses and vegetables (Moye et al., 2018).

As might have been expected, the development of bacterial resistance to the phage during treatment has been a huge potential problem although it does not always arise (Bhandare et al., 2018). However, the problem can be circumvented by:

- Using two phages, one targeting the pathogen and the other targeting the resistant mutants that arise in response to the first phage (Smith and Huggins, 1983), or
- Selecting phages for which the receptors are bacterial surface virulence determinants. In this second case, phage resistance results from loss or alteration of the phage receptor, rendering them less virulent or avirulent (Smith and Huggins, 1982). This latter approach has been adapted for specific use against plasmid-mediated AMR (see immediately below).

In addition to using phages which can target AMR bacteria irrespective of their resistance status, bacteriophages may also be used specifically against AMR bacteria by targeting the mechanism of resistance or of its transmissibility.

In the case of the former, Chan et al. (2016) used a *Myoviridae* (*Caudoviricetes*) phage, for which the receptor was the OprM porin from the MexAB/MexXY drug efflux pump system in a multi-drug-resistant strain of *Pseudomonas aeruginosa*. Phage resistant mutants developed following phage activity *in vitro*. This action resulted in increased susceptibility to a range of antibiotics, resistance to which was mediated by efflux activity.

Two groups have studied the evolutionary effect of using sex pilus-(male-)specific lytic phages which attach to the sex pili of plasmid-containing bacteria. This is a very interesting area of infection biology since, in addition to antibiotic resistance, transmissible plasmid-mediated characteristics include virulence determinants in *E. coli* such as colicin V, *E. coli* enterotoxin, the adhesins K88 and K99, haemolysin and the *Salmonella* virulence plasmid. More recently, the self-transmissible plasmid of emerging *Salmonella* Infantis (pESI) has caused extensive problems in the broiler chickens in Europe and elsewhere (Aviv et al., 2013). This megaplasmid frequently contains genes conferring resistance to multiple antimicrobials along with metals and biocides (Aviv et al., 2013). This opens a potentially wide area of research on the relationship between phages, pathogens, AMR and virulence determinants. Incubation of these phages with AMR bacteria where AMR is highly self-transmissible leads to selection of random plasmid-free variants which come to dominate the culture as the AMR cells are killed by the phages. This has been demonstrated with Tectiviruses and Emesviruses using de-repressed plasmids *in vitro* and *in vivo* with the larvae of the moth *Trichoplusia ni* (Jalasvuori et al., 2011; Mikonranta et al., 2019) and in chickens (Colom et al., 2019).

In reality, most wild type plasmids are at least partially repressed with a small minority of bacterial cells being phage susceptible although contact with lytic phage can actually increase plasmid loss. Unpublished studies with Inc. F AMR plasmids suggest that a pharmaceutical approach to de-repression by blocking the suppressing activity of the FinO protein with pharmaceutically active small molecules might increase phage susceptibility but this has not been developed further. F plasmids dominate in human medicine and are an important Inc. type in veterinary medicine so such an approach might be productive.

There are several advantages to this strategy, particularly since, (i) unlike conventional phage therapy, the phage-resistant mutants are actively selected: (ii) In the absence of the antibiotic to which the bacteria are resistant, the plasmid-free mutants multiply faster than cells containing the plasmid and come to dominate cultures, (iii) The phages are plasmid specific and able to target other bacterial species and different strains harbouring the same plasmid Inc. type, (iv) the lytic phages also kill the AMR bacteria but do not affect AMS bacterial cells, (v) the phages were shown to virtually eliminate conjugation.

This indicates that phages may be used in more ways than one to tackle AMR bacteria and further work in this area is clearly warranted.

##### Application of phage with chemotherapy

Although lytic phages may be used to reduce the level of carriage of AMR plasmids, they have been used in combination with antibiotics and also, for topical use, with disinfectants, under both experimental and clinical conditions involving synergistic mechanisms which are poorly understood. The uncertainty around this combination therapy implies that considerably more research is required before a full assessment can be made.

The combination of phages and antibiotics known as Phage-Antibiotic Synergy (PAS) (Liu et al., 2022) has variable efficacy depending very much on the individual bacteria, phages, the antibiotic used and timing of administration of both (Liu et al., 2022; Fungo et al., 2023; Lin et al., 2021; Lu and Collins, 2009; Wang et al., 2020; Torres-Barceló et al., 2014) with the result that there is as yet no consensus on its value. Recent clues in support of PAS emerged from the statistical analysis of 100 consecutive phage therapy cases in patients, which indicated that bacterial eradication was 70% less probable when no concomitant antibiotics were used (Pirnay et al., 2024).

PAS is speculated to occur through several mechanisms including improving biofilm penetration (Tagliaferri et al., 2019), and bacterial filamentation—particularly relevant with β-lactams and DNA synthesis inhibitors, where the increased cell surface area can increase phage adsorption to each bacterial cell. Additionally, the larger intracellular space may provide greater accumulation of macromolecular building blocks, accelerating phage particle assembly, while more cellular space can also increase burst size (Liu et al., 2022).

However, phage-antibiotic antagonism has also been reported (Zuo et al., 2021). Clearly there remains a great deal to do to determine whether these effects are real and what parameters are involved in defining synergy or antagonism.

#### Node 3b—phage-derived and other products

Endolysins (phage-derived enzymes that degrade bacterial cell walls) have shown therapeutic potential against antimicrobial-resistant Gram-positive pathogens (reviewed extensively by Gondil et al., 2020). Tailocins are bacterial structures produced by several Gram negative and Gram positive bacteria, resembling phage tails that are employed ecologically by bacteria for competitive advantage. They produce needle-like structures which penetrate adjacent bacterial cells depolarising the surface membrane and killing the target bacterium (Backman et al., 2024). They can be engineered from bacteriophages (Woudstra et al., 2023), but their specificity may limit their exploitation and their use currently remains at the experimental stage.

Studies have explored combining these agents for enhanced antimicrobial effects. Lu et al. (2023) reported synergistic effects when bacteriophages, endolysins, and antibiotics were used together against pathogenic *Shigella flexneri*. The greatest synergy was observed with cefotaxime (β- lactam antibiotic) combined with lysis proteins (LysSSE1 or HolSSE1). Gouveia et al. (2022) demonstrated that synthetic antimicrobial peptides (AMPs) can improve the bacteriolytic action of staphylococcal phage endolysins against *S. aureus*, including drug-resistant clinical isolates.

Combinations of plant extracts and bacteriophage have been tested *in vitro* and *ex vivo*, but not yet on clinical animal trials. Stachurska et al. (2023) investigated interactions between lytic phages and *Stevia rebaudiana* extracts, finding that their effects varied depending on bacterial species, and for phage MS2 the plant extract proved to be viricidal. Pimchan et al. (2018) observed that phages combined with plant extracts significantly reduced bacterial numbers in the short term, though not more effectively than phages alone after 24 h.

To target Gram-negative pathogens, some endolysins possess an intrinsic ability to partially disrupt the outer membrane; however, in most cases, engineering strategies or combination therapies are required to enhance membrane penetration (de Maesschalk et al., 2020). One such approach involves the use of antibiotics such as colistin, which permeabilize the outer membrane and thereby enable endolysins to access and degrade their peptidoglycan substrate (Rothong et al., 2024). Moreover, persistent *A. baumannii* strains following tobramycin treatment could be successfully targeted by designer endolysin Art-175, resulting in a clear synergistic action (Defraine et al., 2016).

Combination therapy has also been attempted using phage with other biocontrol agents. Hobley et al. (2020) showed that lytic bacteriophage used in combination with the predatory bacterium *Bdellovibrio bacteriovorus* resulted in a synergistic reduction in *E. coli* prey *in vitro*. Conversely, cultures exposed only to *Bdellovibrio* were reduced by over 2 log_10_ CFU/mL over 48 h but at a slower rate than phage infection. The combination of both agents resulted in a rapid decline in the bacterial population, below detectable limits, and without recovery of resistant subpopulations. Chen and Williams (2012) reported a similar co-infection/predation of the pathogen *Vibrio vulnificus* in what the authors termed a “competitive alliance.” The benefits of this type of combination therapy will likely only be realised in a limited range of applications, as *Bdellovibrio* are more rapidly removed by the immune system of vertebrate animals than phage, based on available reports (Willis et al., 2016; Capparelli et al., 2006). Moreover, the trade-offs of this alliance will likely vary depending on the particular combination of phage(s) used with *Bdellovibrio*. Model lytic phage such as T4 have been found to outcompete *Bdellovibrio* in continuous culture in all circumstances (Summers and Kreft, 2022).

As with PAS the use of these other biological agents are clearly in the experimental phase and until they have been studied in great depth no further recommendations for their use in a clinical setting can be made.

### Node 2—Phage as delivery vehicles

We discuss here briefly the potential for bacteriophages as delivery platforms to facilitate bacterial genome manipulation and for the delivery of other therapeutics.

#### Node 2a— bacterial genome editing

Within the context of phage therapy, the adoption of genome editing tools for both phages and their bacterial host has huge potential for modulating bacterial geno/phenotype including AMR and virulence.

Phage genomes may be manipulated such that novel genes and associated regulatory elements are introduced as genetic cargo to achieve desired changes to bacterial phenotypes (Chen et al., 2019; Du et al., 2003). Our increasing understanding of phage genomes and genetics, combined with the small size of some phages, makes them tractable to genetic engineering.

There is increasing evidence for the successful use of phage-based vectors for the systemic delivery of therapeutic genes in cancer therapy and other clinical problems (Serwer and Wright, 2018). Bacteriophages show greater efficiency of transgene delivery and expression in cancer cells compared to non-viral gene transfer methods. In comparison with eukaryotic viruses, phage-based vectors also have a greater margin of safety for delivery to mammalian cells (Petrov et al., 2022). Phage have been used to deliver CRISPR-associated transposases to target bacteria resulting in large genome deletions (Roberts et al., 2025).

Strictly lytic phages, which are preferred for phage therapy, can only be engineered *in vivo* during the brief infection cycle which poses a critical hurdle compared to temperate phages or bacterial lysogens. As such, this genome engineering will rely on efficient homologous recombination. The established Bacteriophage Recombineering of Electroporated DNA (BRED) approach is a key example of this, relying on linear phage DNA and synthetic DNA with the desired mutation that are co-introduced into the bacterial host cell expressing phage recombination genes. The presence of these phage-derived recombinases results in an improved efficacy of recombination (Marinelli et al., 2008). Recent alternatives combine homologous recombination with specific CRISPR-Cas systems, providing a more versatile, rapid and low-cost approach. Utilizing the bacterial adaptive immune system to introduce double-strand breaks by a single guide-RNA in the target DNA in the presence of a repair template containing the desired mutation (deletions, insertions or substitutions) has proven to be widely applicable for phage engineering or may serve as a counter-selection method for the wildtype phage.

The most-studied CRISPR-Cas9 system has been shown to edit phage genomes infecting *Vibrio*, lactococci, *Klebsiella*, *Mycobacterium*, *E. coli*, *Listeria* and *Streptococcus*. By contrast, the CRISPR-Cas3 system, introducing larger deletions, proved more efficient in *Pseudomonas* species (Lammens et al., 2023) and proves beneficial for genome-scale deletions of the production host. However, some phages circumvent DNA-targeting immunity, by carrying anti-CRISPR proteins, DNA base modifications, or genome segregation. For example, when editing giant phages like *Pseudomonas* phage phiKZ, which builds a nucleus structure during their replication cycle, a tailored solution based on the mRNA targeting CRISPR-Cas13a system is required to provide a robust selection mechanism (Guan et al., 2022).

#### Node 2b—delivery of therapeutics/vaccines

The ease of phage manipulation has resulted in several related approaches to the use of phage capsids as potential vaccines using phage display as a means to present antigens and epitopes on the phage surface (Clark and March, 2006). Several different phages have been used against a variety of bacterial (plague, anthrax) and viral (influenza, foot and mouth disease, and hepatitis B) infections and different cancers (Hess and Jewell, 2019). This approach is also amenable to the development of diagnostic assays (Chen et al., 2025). Phages have also been used for the delivery of therapeutics like intrabodies to target plasmid relaxases affecting plasmid stability (Garcillán-Barcia, et al., 2007) or antimicrobial peptides (AMPs). Their use to deliver pharmaceutically active molecules to reduce neural inflammation offers exciting opportunities for other non-infectious conditions (Zhu et al., 2025).

### Node 1—Phage technology for control of pathogens and AMR: delivery, storage, field trials, safety, regulation

Universal uptake of phage in agriculture and veterinary and One-Health medicine is dependent on demonstration of efficacy, ideally in the field or under experimental conditions that reflect closely field conditions. The state of the art covered in this road-map shows how variable the results can be from research using field infections or experimental infections mirroring closely field conditions, dependent as the results are on so many factors, even though many will be controllable.

Critical aspects for effective use of phage technology such as developing robust protocols for assessing safety of the phage product, studying optimal delivery route (e.g., oral, topical, injectable) and suitable delivery platforms and demonstrating efficacy in challenge models falls into Node 1. This includes overcoming challenges of proving efficacy and safety of biological entities in compliance with current regulations. Regulatory authorities face challenges with biological entities as most existing legislation relates to chemical antimicrobials. Further problems arise from the quantity and quality of data on which to produce a defined regulatory framework. Intellectual property is a further hurdle which phage banks may overcome.

### Technical aspects—approaches to delivery and storage

Further progress needs to be made on the practical aspects of phage storage and delivery (Malik et al., 2021). A degree of knowledge of the pharmacodynamics of the phage under storage is vital to ensure that as high a titre as possible is delivered to the right part of the host without damage to the phage DNA/RNA or the capsid proteins.

Phage preparations may be freeze-dried, spray-dried or spray-freeze-dried for storage. Encapsulation in micro-or nano-particles (Lorenzo-Rebenaque et al., 2021) may be used with natural or synthetic polymers to which may be added sugars such as sucrose or trehalose to increase phage survival, particularly in the gut (Zhang et al., 2023; Dlamini et al., 2023). Additional surface modifications may be made to provide additional specific biological properties. The choice of polymer and chemistry is complex and may be made for specific reasons, for example to protect against low pH in the gut (Colom et al., 2015). Liposome formation can increase accumulation in macrophages thereby opening the possibility of targeting intra-cellular bacterial pathogens. The use of liposomes may also facilitate biofilm penetration and may be positively charged to associate more closely with mucosal membranes (Ibaraki et al., 2020). These issues are important to delivery to sites where glycans may inactivate unprotected phages.

Areas for future exploration include membrane emulsification, the use of microfluidics and polymers which can trigger phage release on stimulation with environmental signals present in the body of the target host (Malik et al., 2021; Malik and Resch, 2020). Maintenance of phage titre may also be regulated by delayed release into the more distal sections of the gut (Vinner et al., 2019).

Protection against the adaptive immune response will be particularly important where protracted treatment and phage delivery is being considered (Berkson et al., 2024) possibly requiring multiple high titre dosing. Liver sinusoid cells can remove phage from the circulation soon after intra-venous administration although for some septicaemias the circulation of bacteria between the blood and liver provides sufficient circulating target bacteria cells for phage therapy to be effective (Barrow et al., 1998). In one study, circulating antibodies against phage were not found following oral phage administration (Berchieri et al., 1991).

### Technical issues—use in the field—farm trials

Phages may be currently delivered to the body by a variety of routes dependent on the nature of the infection, preferred administration routine, knowledge of phage longevity *in vivo* and convenience. They must remain viable in sufficient numbers to be effective and thus must overcome the various components of innate immunity presented by the host in the tissues and alimentary tract.

The literature on phage application using animal models of infection is large and complex and sometimes confusing because the requirements for delivery may vary according to the pathogen and circumstances. Oral administration of phage understandably requires protection against low pH. Antacids may be administered prior to or simultaneously with phage. For convenience, delivery in feed or drinking water might be considered but the additional factors of survival in the food and water must then be considered. For respiratory infections, nasal spray is the most appropriate route for delivery but may not deliver the highest phage titre and a parenteral route, such as intra-peritoneal or intra-muscular, might be more effective at delivering higher titres of phage at the infection site. This might also be the case for some wound infections. Treatment of extra-cellular systemic infections such as sepsis, would appear to be straight forward and require intra-venous delivery but an intra-cellular element is also involved in such infections and although delivery of phage to intra-cellular niches has traditionally been considered extremely difficult, recent approaches may improve this in the longer term. The problems of application to different infections reflect the characteristics and problems of the unique individual infection types, whether this be the impact of complex microflora in the lower intestine, respiratory mucus in cystic fibrosis and chronic intra-cellular bacterial infections such as tuberculosis.

As with many biological products the effectiveness assessed *in vivo* under controlled laboratory conditions are not always replicated in the field sometimes because of animal management and other factors but also because of additional biological imponderables present in the field. Consistency in data is important and for some infections a solution to this might be worth standardizing the model for each major infection type, namely: gastro-intestinal, respiratory, sepsis and skin infections.

A large literature exists demonstrating considerable efficacy of phages in poultry and also pigs under very controlled conditions reducing the numbers of enteric bacteria such as *Salmonella* (Atterbury et al., 2007; Nabil et al., 2024; Pelyuntha et al., 2024; Thanki et al., 2022) and *Campylobacter* (Loc Carrillo et al., 2005; Peh et al., 2023). There have been few reports demonstrating efficacy in the field. This might be expected since on-farm assessments of phage use against enteric infections are complicated by the frequent multiplicity of pathogens, bacteria, viruses and sporozoans involved. A commercial preparation SalmoFREE^®^ (a previously genomically and phenotypically characterized mixture of six *Salmonella* lytic bacteriophages) was effective against *Salmonella* in one trial in Columbia but not in another (Clavijo et al., 2019). In Germany, a two-phage mixture tested against *Campylobacter* in broilers led to a reduction in bacterial numbers in the faeces after one application but not after another (Bogun et al., 2024). Assessment in young calves with diarrhoea given suppositories of three phages but together with *Lactobacillus* spp. reduced the number of clinical signs and rectal temperatures (Alomari et al., 2021). The involvement of mixed infections was also problem in treatment of superficial pyoderma in horses where a reduction in the target pathogen *S. aureus* followed treatment with two phages but overgrowth by other cocci resulted in no clinical improvement (Marshall and Marsella, 2023).

A single phage (Lerondelle and Poutrel, 1980), thought by others to be ineffective, and assessed against *S. aureus* mastitis, yielded small but statistically insignificant improvements in mammary gland health (Gill et al., 2006).

As far as we know no results have been published of extensive field trials on the effect of phages against fish diseases although there is evidence of effectiveness under strictly controlled conditions (Nakai et al., 1999; Nokhwal et al., 2021; Schulz et al., 2019a; Schulz et al., 2019b).

In companion animals, interest in therapy has been mainly focused on treatment of canine otitis with a published report reporting good short- and long-term improvements in clinical score and bacterial load following administration of a six-phages mixture (Hawkins et al., 2010).

The relatively few trials at the farm level that have been published have involved different animal species, diverse administration routes, including oral, rectal, intramammary, topical, and injectable methods, as well as various phage formulations. Such field trials raise concerns over the initial trial planning including selection of the appropriate phage(s) and field conditions associated with these trials. Overall assessments are therefore difficult and the variation in the results produced indicate the importance of biological variability (Malik et al., 2023). Clearly, there must be considerable merit in establishing standard protocols for field trials with specific pathogens. Better-controlled trials are required to demonstrate efficacy and give confidence that on balance, even in the field with the multiple factors that may affect results, specific phage administration can be a positive benefit in the majority of cases.

### Technical issues—regulatory issues including safety

The lack of a defined regulatory framework was regarded as a key reason for the lack of investment in phage therapy in some countries until recently (Hodges and Smith, 2024). This was despite phage-derived treatments listed among the most promising alternatives to treat antimicrobial-resistant infections (Czaplewski et al., 2016). This was partly due to existing standards for therapeutic agents being based upon antimicrobial chemotherapy which do not easily lend themselves to bacteriophage. A paucity of high-quality data – particularly from large-scale trials—could also limit the ability of regulators to approve new treatments. Moreover, the breadth of available bacteriophage-derived therapies poses further difficulties in that phage, or their components (e.g., endolysins, tailocins etc) have been used both natively and in genetically modified forms. As such, although there may not be any regulatory barrier to authorising phage-based products *per se*, further clarification from regulators may be required to guide innovators through dedicated approval pathways.

Regulation may be further complicated by limited and sometimes contradictory data on the reproducibility of results from phage therapy trials (Miedzybrodzki et al., 2023). This may result from several factors as indicated above. In the UK, there have been movements to address this in part through the development of phage reference reagents and standardised methodologies based on previous guidance produced for the development of microbiome products (Hodges and Smith, 2024).

In Belgium, France and more recently Portugal, hospital and academic facilities now provide phage therapy solutions through magistral preparation of personalized phage treatments. This approach is put forward in parallel to the establishment of traditional drug development pathways for fixed phage cocktails under GMP production. The magistral preparation of phage refers to the small-scale, personalized formulation phages by hospital or academic pharmacy facilities. These preparations are tailored to individual patients based on their specific bacterial infections. Unlike commercially manufactured phage products, magistral preparations are not mass-produced but are instead compounded on demand, following medical prescriptions by the treating physician (Pirnay et al., 2018; Pirnay and Verbeken, 2023). While useful to treat dozens to hundreds of patients, it remains unclear how this approach could be scaled-up, while retaining the quality requirements set forward in individual countries.

Large scale industry-funded investment in phage therapy has also been hampered by intellectual property concerns, which are often intertwined with regulatory and enforcement hurdles. Bacteriophage therapy is not a new idea, having first been explored more than 100 years ago (d’Herelle, 1921). Despite this, patent protection has been—and continues to be—granted for phage-based therapeutics. In 2024, a global search for patents containing “phage therapy” results in over 1,155 records^9^ which is more than double the number reported just 3 years before (MacLean and Harper, 2020). Patenting of natural, unmodified phages is challenging in some jurisdictions such as the United States, which stems from the “Myriad judgement” of the US Supreme Court which concluded that “products of nature” were not patentable (MacLean and Harper, 2020). This does not prevent patenting *per se*, provided that the inventor can demonstrate a discernibly different property not present in the natural product. Patenting of genetically modified bacteriophage products – for example with extended host ranges or employing the CRISPR-Cas system—is likely to be more straightforward. However, this approach may encounter far more significant regulatory and enforcement hurdles involving the release of self-replicating GM agents into the environment. A further approach has been to patent novel processes or technologies related to optimising or purifying bacteriophage. However, even if such patents are granted, inventors may still be prevented from commercialising their products by the existence of third-party patents which may block implementation. This is often addressed through a “freedom-to-operate” search which focusses on prior patents (not limited to bacteriophage therapy specifically) that may present a barrier to market (MacLean and Harper, 2020).

### Technical issues—phage banks

One approach which may facilitate phage product regulation is the use of pre-characterised collections of phages or ‘phage banks’ (Nagel et al., 2022). More recently, phages for Global Health (PfGH) initiated a project to draft phage biobanking guidelines through consultation with the global phage community (Nagel et al., 2022). The UK Phage Innovation Network was invited to collaborate, along with Phage Canada, Phage Australia and Phage Directory to develop recommendations on phenotypic and genotypic analysis of phage as well as physical storage and data management (Hodges and Smith, 2024). National or regional phage banks could not only facilitate regulation but also act as a resource for the rapid and controlled deployment of phage to address outbreaks of antibiotic-resistant infections (Nagel et al., 2022). To date, phage banks have been focussed mainly on the treatment of bacterial infections in humans. The main obstacle to extending this practice to animal diseases is the cost of infrastructure to maintain and manage these collections, which is more easily justified if the focus is on human patients. Some phage banks in Eastern Europe and the former USSR have been used in the preparation of pre-set phage cocktails for wider distribution to human patients (Nagel et al., 2022), in contrast to the more individualised approach reported in human phage therapy trials in the West (Abedon et al., 2011; Pirnay et al., 2024). While these pre-set formulations may be more compatible with livestock and agricultural applications of phage therapy, such products require regular testing and reformulation in order to maintain efficacy. As such, resources of this kind are likely to require government- or a public-private funded partnership to be viable, given the relatively low margins in the agricultural and livestock sectors.

An increasing number of phage banks are being established worldwide, and these collections range in size from a few hundred to many thousand phages (Yerushalmy et al., 2020). Large and longstanding collections of phages are available from the ATCC (USA), NCTC (UK) and Felix d’Herelle Reference Centre for Bacterial Viruses (Canada) as well as the SEA-PHAGES programme at the University of Pittsburgh. Established clinically-focussed collections include the Eliava Institute of Bacteriophages, Microbiology and Virology in Georgia; and the Hirszfeld Institute of Immunology and Experimental Therapy in Poland. If phage banks are to be deployed safely across different regulatory areas, it follows that harmonisation of standards on storage, quality assurance and data management would be required; ideally along with a well-characterised bank of bacterial host strains and their AMR status (Nagel et al., 2022; Wahid, 2024). Further benefits could be derived from sharing metadata on the efficacy of phages in different species or environments (such as biofilm vs. planktonic cells) or ability to target specific receptors which may be associated with antimicrobial resistance or virulence (Wahid, 2024).

A means whereby phage therapy could be seamlessly integrated into current veterinary and medical practices will necessarily involve extensive collaboration between regulatory bodies, researchers, and industry stakeholders. This multi-faceted approach is crucial for developing scalable, effective, and consistent phage-based treatments which can address the growing threat of AMR. By investing in advanced molecular techniques and fostering international cooperation, the potential of phage therapy can be harnessed to its fullest extent. The establishment of robust phage banks, coupled with standardized protocols and thorough regulatory frameworks, will pave the way for the successful deployment of phage therapy across various sectors.

### Summary—the future and road map

It is clear that, although bacteriophages have been mooted as valid antibacterials for more than 100 years, and despite the fact that they have been used and continue to be used in many countries, the current period almost seems like a reset involving a completely objective reassessment and evaluation of natural phages, both as antibacterials and as a means to specifically tackle the existential problems of AMR. Clinical, biological and administrative issues and ways forward are outlined in this review and highlighted in the roadmap. One important feature is the huge amount of work that has already been carried out using experimental animal models and field trials but also on encapsulation for delivery and storage. A degree of standardisation could expedite further development and progress in each of these areas.

From the expansive discussion of some of the above nodes in this exploration of research needs the STAR-IDAZ group has identified the following key points requiring substantial research and investment to enable the technology to make further progress:

- The nature of phage-bacteria interactions for the phage classes that are or might be used.
- *In vivo* models and trials using the most appropriate models.
- An investigation of phage survival in the animal and in the environment.
- A detailed exploration of synthetic biology for retargetable phage-based platforms.
- Interaction between phage and the immune system both in terms of persistence *in vivo* but also of the synergy between both during therapy.
- High throughput screening platforms for phage isolation/characterisation and the exploration of machine learning and AI in the filtration process.

Some of these difficult technical, practical and regulatory issues are set against the huge technological opportunities afforded by the advances made in AI, molecular biology and genetic manipulation, both to modify the phages themselves and to explore phages for delivery of nucleic acid and proteins to modulate AMR, virulence and disease and to which the proposed research areas for future exploration above will contribute. Despite the huge problem of AMR confronted by society it is clearly an exciting time to be working with bacteriophages as antimicrobials.

## Footnotes

1
https://eliava-institute.org

2
https://eptc.ge

3 www.fao.org/georgia/news/detail-events/en/c/1652530; www.fao.org/georgia/news/detail-events/ar/c/1627284/

4
https://www.who.int/news/item/01-12-2021-tripartite-and-unep-support-ohhlep-s-definition-of-one-health

5
www.woah.org/en/what-we-do/global-initiatives/antimicrobial-resistance/

6
www.who.int/news-room/fact-sheets/detail/antimicrobial-resistance

7
www.star-idaz.net

8
https://www.star-idaz.net/roadmap/roadmap-for-phage-technologies/

9
https://worldwide.espacenet.com/patent/
